# Managing Early Onset Osteoporosis: The Impact of Premature Ovarian Insufficiency on Bone Health

**DOI:** 10.3390/jcm12124042

**Published:** 2023-06-14

**Authors:** Blazej Meczekalski, Olga Niwczyk, Gregory Bala, Anna Szeliga

**Affiliations:** 1 Department of Gynecological Endocrinology, Poznan University of Medical Sciences, 60-535 Poznan, Poland; olga.niwczyk@gmail.com (O.N.); annamaria.szeliga@gmail.com (A.S.); 2 UCD School of Medicine, University College Dublin, D04 V1W8 Dublin, Ireland; greg.bala1@gmail.com

**Keywords:** POI, hormonal replacement therapy, testosterone therapy, BMD, osteoporosis

## Abstract

Premature ovarian insufficiency is a reproductive endocrine disorder characterized by the cessation of ovarian function before the age of 40 years. Although the etiopathology of POI remains largely unknown, certain causative factors have been identified. Individuals affected by POI are at an increased risk of experiencing bone mineral density (BMD) loss. Hormonal replacement therapy (HRT) is recommended for patients with POI to mitigate the risk of decreased BMD, starting from the time of diagnosis until reaching the average age of natural menopause. Various studies have compared the dose-effect relationship of estradiol supplementation, as well as different HRT formulations on BMD. The impact of oral contraception on reduced BMD or the potential benefits of adding testosterone to estrogen replacement therapy are still subjects of ongoing discussion. This review provides an overview of the latest advancements in the diagnosis, evaluation, and treatment of POI as it relates to BMD loss.

## 1. What Is Premature Ovarian Insufficiency?

Primary ovarian insufficiency is a reproductive endocrine disorder characterized by cessation of ovarian function before the age of 40 years. The gold standard for establishing a diagnosis of POI is the assessment of follicle stimulating hormone (FSH). The European Society of Human Reproduction and Embryology (ESHRE) guidelines [1] recommend a diagnosis of POI when an elevated FSH level > 25 IU/L is recorded twice at least four weeks apart in the setting of oligo/amenorrhea lasting at least four months in women under 40 years old. In patients with POI, there is a notable decrease in bone mineral density (BMD) attributed to inadequate accrual of peak bone mass. This is primarily caused by hypoestrogenism and hypoandrogenemia in young women with POI. Additionally, the decrease in BMD is linked to increased bone remodeling, particularly bone resorption.

## 2. Etiopathogenesis of POI

Although most instances of POI have an unknown etiopathology, certain causative factors have been established. Genetic predisposition and chromosomal abnormalities are both common causes of primary ovarian insufficiency. Kalantari et al. [2] observed that the overall prevalence of chromosomal abnormalities in a group of 179 Iranian women with POI was 10%. Turner’s syndrome, especially cases of 46XX-dominant mosaicism, is the most common chromosomal abnormality associated with POI, with over 50 genes identified as having involvement in the condition. Among genetic causes, mutation of the fragile-X mental-retardation 1 (FMR1) gene, causing Fragile-X syndrome, warrants particular attention. Evidence has emerged that women who carry the premutation allele have up to a 13% increase in risk of developing POI [3].

The association between autoimmune disease and POI has been investigated in many studies. It is estimated that 4–30% of POI cases are autoimmune in origin. POI of autoimmune origin can be divided into three types: adrenal autoimmunity, non-adrenal autoimmunity, or isolated [4]. POI of adrenal autoimmune origin is the most frequent type, observed in 60–80% of cases. Non-adrenal autoimmunity can be related to thyroid diseases, hypoparathyroidism, and type-1 diabetes mellitus, as well as non-endocrine autoimmune diseases such as systemic lupus erythematosus or rheumatoid arthritis. ESHRE guidelines recommend screening for thyroid TPO-Ab antibodies and 21-OH-Ab or adrenocortical antibodies (ACA) in women with POI of unknown cause.

Patients after radio- and chemotherapy, as well as patients after ovarian surgeries, are at risk for iatrogenic POI. As it relates to ovarian damage, the effect of chemotherapy depends largely on the drug, dose, and patient age. The effect of radiotherapy, on the other hand, is mainly a product of therapy field, with abdominopelvic radiation being most likely to result in irreversible ovarian damage [5].

Many case reports have documented viral infections (including mumps, human immunodeficiency virus, and cytomegalovirus) antecedent to POI, however no case has yet directly established infection as contributory to POI pathogenesis [6,7]. As a result, infection screening is not recommended in women with POI [1].

Smoking, alcohol intake, nutrition, and other environmental factors are all considered contributory to the age of menopause onset but are not by their presence alone diagnosable causes of POI [8]. Despite the ongoing progress in the field of menopause research, most cases of POI have no identifiable root cause and remain classified as idiopathic.

## 3. Peak Bone Mass (PBM) and Bone Mineral Density (BMD)

Peak bone mass (PBM) refers to the maximum amount of bone tissue present at the end of skeletal maturation [9]. It is crucial to achieve PBM during childhood to prevent the risk of osteoporosis in later life. Although the age at which peak bone mass is attained may vary individually, about 85–90% of final adult bone mass is acquired by the age of 18 years in girls and 20 years in boys [9]. Several factors interfere with gains in bone density, with race, gender, and genetics being the most crucial. Zhai et al. [10], explored and documented the significant weight that genetics carry in determining PBM when they studied a group of 712 postmenopausal Caucasian women to establish the heritability of bone loss in humans. Their data suggest that 56% of the inter-individual variance in bone loss was genetics-driven [10]. The gender difference in mean PBM observed in healthy young adults can be attributed to the additional year of accelerated bone growth experienced in males: four years vs. three years in females [11]. Additionally, females experience a drastic decline in the rate of bone mass accumulation by the age of 16 years [12]. A variety of lifestyle factors have been linked to PBM, including physical activity, calcium and vitamin D intake among other dietary components, smoking, alcohol consumption, socioeconomic status, age at menarche, and many other secondary causes [13]. Physical activity, for instance, increases bone mineral mass accumulation in both children and adolescents when force applied on the skeletal structure stimulates intracellular processes in bone tissue and mineral deposition in the bone, resulting in an increased BMD [14]. Vitamin D also plays a crucial role in bone mineral mass gain, with early supplementation proving to be beneficial in improving areal bone mineral density (aBMD) in later childhood [15]. In a longitudinal study by Chevalley et al., total calcium intake was shown to have a positive effect on PBM, especially before the onset of puberty [11]. Impaired bone growth can often be observed in correlation with different health conditions such as rheumatoid arthritis, inflammatory bowel diseases (IBD), and anorexia nervosa, for instance. Adolescents with IBD tend to have lower PBM and altered trabecular bone microarchitecture compared with healthy peers [16]. Anorexia nervosa not only decreases rates of bone accrual compared with normal-weight controls but ultimately leads to suboptimal peak bone mass.

Bone mineral density assessment of the femoral neck and lumbar spine is considered the gold standard for evaluating osteoporosis [17]. Dual-energy X-ray absorptiometry (DXA) is the established reference technique for measuring BMD. Prospective studies have consistently shown that low BMD, and subsequently osteoporosis, is a key factor in determining the risk of fragility fractures. As BMD decreases, and the risk of fractures increases, BMD and DXA measurements can function as predictors of this risk [18]. It is important to note that while BMD is a major determinant of fracture risk, it is not the only one. By combining BMD with other risk factors such as age and prior fracture history, a more accurate assessment of an individual patient’s fracture risk can be achieved, surpassing the predictive ability of bone density alone [19]. The FRAX algorithm, developed by the World Health Organization (WHO), integrates the influence of multiple validated risk factors and reports the 10-year probability of hip or other major osteoporotic fracture. However, it is worth noting that this tool has not been validated for women under the age of 40 years.

## 4. Estrogens and Their Influence on Bone Metabolism

Estrogens play a critical role regulating bone turnover in both females and males. Since the ground-breaking study by Lindsay et al. [20] in 1976, which demonstrated the preventive effect of synthetic estrogen therapy on bone loss in oophorectomized women, much research has focused on understanding the mechanisms by which estrogen regulates bone metabolism. Estrogen effects are mediated by two receptors, estrogen receptors alpha (ERα) and beta (ERβ), both of which have been detected on osteoblasts, osteocytes, and osteoclasts [21].

Estrogens reduce osteoclastogenesis and stimulate osteoclast apoptosis. Osteoclast-selective deletion of ERα causes an increase in osteoclast progenitor development in the marrow (followed by increased osteoclast numbers), and subsequently decreased trabecular bone mass [22]. Estrogens reduce human osteoclast formation via ERα activation, suppressing receptor activator of NF-κB ligand (RANKL) and impairing RANKL-induced osteoclastogenesis [23]. In addition to these direct pathways, estrogen appears to indirectly affect osteoclasts through repression of pro-osteoclastic cytokines such as IL-1, IL-6, IL-7, and tumor necrosis factor (TNF) [24]. Estrogens have been shown to inhibit osteoblast apoptosis and increase osteoblast longevity by repressing apoptotic gene expression and inhibiting caspase-3/7, which plays a central role in coordinating apoptosis [25].

Several studies have demonstrated the significant role of bone cell ERα in bone adaptation to mechanical strains, as ERα is involved in the osteogenic effects of loading. Animal models comparing mice lacking ERα or ERβ activity have shown that ERα enhances the net-osteogenic response to loading in cortical (but not cancellous) bone, whereas ERβ decreases it [26]. It is believed that estrogens may, in part, regulate bone mass as a secondary effect to its regulation of skeletal muscle mass and contractile function. ERα deletion, but not ERβ deletion, reduces mass and contractile properties of some muscles in mice [27]. As age increases, the number of oxidative stress products in bone also increases, which can attenuate osteoblastogenesis. Estrogens inhibit 15-lipoxygenase, the enzyme responsible for the generation of oxidized low-density lipoprotein, partially preventing OVX-induced bone loss [28]. Yet another postulated mechanism of estrogen action is estrogen-mediated suppression of sclerostin production, the potent inhibitor of bone formation. Estrogen treatment of postmenopausal women resulted in a decrease in circulating sclerostin levels [29].

## 5. Prolonged Hypoestrogenism and Other Risk Factors for Decreased BMD and Increased Fracture Risk

The first mention of POI traces back to Fuller Albright, who in 1941 described many aspects of the disease, such as the complex etiopathogenesis of decreased bone mineral density (BMD) [30]. Albright was the first person to present a scientific publication in which they linked estrogen deficiency and menopause with increased risk and incidence of bone fractures.

Patients with POI are vulnerable to bone mineral density loss (osteopenia and osteoporosis) [31,32]. The estimated prevalence of low bone mass in POI ranges from 8–27%, depending on the definition used and causative factor of POI [31,32]. Both the prevalence of osteoporosis and fracture risk in women with POI is double that of the general population, with the rate of osteoporosis being double that of women after natural menopause [33].

A main question which currently stands to be addressed is that of the underlying cause of decreased BMD and increased fracture risk in women with POI. Many causes have been identified, but two feature most prominently among them.

Firstly, patients with POI present low BMD due to insufficient peak bone mass accrual [34]. Hypoestrogenism and hypoandrogenemia in young women with POI can contribute to this process. Secondly, an increase in bone remodelling, resulting mainly in bone resorption, ultimately leads to a decreased BMD. This bone resorption develops secondary to estrogen deficiency [35]. The most important risk factors contributing to BMD loss in POI are the degree and duration of estrogen deficiency [34]. The type of bone which is susceptible to loss is also characteristic of POI, with greater loss of trabecular bone than cortical bone [1]. However, the role of trabecular bone versus cortical bone in determining strength at the femoral neck appears to be minimal, indicating that changes in trabecular bone and their impact on fracture risk are relatively insignificant [36]. POI after iatrogenic treatment is also associated with decreased BMD. Chemotherapy in young age can contribute to the development of POI and an associated decrease in BMD. Patients with POI following chemotherapy for gynaecological malignancies were found to have a significantly decreased BMD when compared to controls (39% vs. 15%, respectively; *p* = 0.009) [37]. This decrease in BMD is mainly attributed to estrogen deficiency.

High serum FSH levels seem to have a negative impact on BMD [38]. Lana et al. [39] have reported that in patients with spontaneous POI serum FSH concentrations, but not estradiol, are positively associated with bone mass loss in the skeletal regions of both the spinal column and femoral neck in patients with spontaneous POI. Patients with POI are characterized by a significant decrease in serum androgen levels (hypoandrogenemia). Androgens have a positive impact on PBM formation and BMD formation [40]. It should be emphasized that bone status is significantly related to muscle status. A decrease in the levels of estrogen and androgen contributes to a decline in muscle mass, which, in turn, can cause a decrease in BMD. Although no study has yet been performed in which this mechanism is substantiated in women with POI, both estrogen and androgen receptors have been found expressed on myocytes. The action of estrogen on muscle metabolism and homeostasis is well known, while the action of androgens lends itself mainly to anabolic effects [41].

Additional risk factors which are related to a decreased BMD in POI include a delay of greater than 1 year in diagnosing POI, vitamin D level below 32 ng/mL, low calcium intake, and lack of physical exercise [42].

## 6. Diagnosis of Osteopenia and Osteoporosis in Women with POI—Insufficient Tools and Clinical Problems

In evaluating bone status in women with POI, it is incredibly important to remain adequately sensitive to detecting bone mineral density (BMD) loss and addressing fracture risk. The strength of bones, and thus the risk of fracture, is influenced by bone mass, geometry, and microarchitecture [43].

At first glance, diagnosing osteopenia and osteoporosis in patients with POI may seem simple and straightforward, but the broader clinical situation is often more complicated than initially apparent. Numerous factors and tools which are normally used to diagnose decreased BMD and stratify fracture risk has limited application in cases of POI.

Dual-energy X-ray absorptiometry (DXA) is the gold standard method used to diagnose osteopenia and osteoporosis. The European Society of Human Reproduction and Embryology (ESHRE) recommends that a DEXA examination should be performed in all young patients with amenorrhea lasting more than 6 months as a result of hypoestrogenism [1]. However, DEXA has some limitations which are particularly important in the context of POI patients, primarily that this method does not differentiate between cortical bone and trabecular bone [44]. This is particularly important, as the relative contribution of trabecular to cortical bone with respect to bone strength in the femoral neck seems to be marginal [36]. Additionally, it does not provide any information on bone quality or geometry, while in patients with short stature, such as in cases of Turner’s syndrome, results need to be adjusted for height [45]. A similar set of problems is faced by patients treated for malignant bone tumors such as osteosarcoma [46]. The relationship between BMD and fracture risk has not been well studied in the group of predominantly young women with POI.

The FRAX method provides a good approach to estimate fracture risk in postmenopausal women. However, this tool has not been validated for women younger than 40 years of age [47] making its applicability questionable for use in most women with POI. Understanding that BMD loss in patients with POI is mainly trabecular, a specific parameter—the trabecular bone score (TBS)—can provide particularly insightful information. This score, introduced in 2008, is a measure of bone texture correlated with bone architecture and a marker for the risk of osteoporosis [41].

Nguyen et al. [48] reported a strong association between low TBS and fracture prevalence in women with Turner’s syndrome. In the absence of more specific tools, TBS is a promising adjunct to DEXA for guiding the evaluation of POI-related bone health; however. it does require further study and validation.

Peripheral quantitative computed tomography (pQCT) and high-resolution pQCT are considered useful methods to assess the difference between trabecular and cortical bone in women with POI. These methods, however, are prohibitively expensive in most cases and not widely available. This method was used by Soucek et al. in their study evaluating bone status in women with TS [49].

## 7. Treating POI

Patients with premature ovarian insufficiency (POI) are at an elevated risk of decreased bone mineral density and should be treated with hormonal replacement therapy (HRT) from the time of diagnosis until the mean age of natural menopause [50,51].

Recent studies have showen that HRT significantly increases bone mineral density (BMD) in the lumbar spine, femoral neck, and total hip [41]. Although the need for HRT is undisputed, evidence for the optimal HRT regimen is lacking, while consensus on dual-energy X-ray absorptiometry (DXA) screening and monitoring varies. Treatment must be adapted to each patient on an individual basis and to each patient’s individual needs. For patients starting on therapy, it is recommended that a follow-up DXA scans of hip and spine be performed after two years, using the same instrument when possible. If BMD is stable or improved, less frequent monitoring can be performed thereafter [52]. Some have argued, however, that if BMD remains normal while on adequate HRT, the added value of performing repeat DXA scans is low. If a low BMD is observed, on the other hand, repeated DXA scans should be performed every 2–5 years [41].

The latest trials on the use of HRT in POI have indicated a positive correlation between treatment and BMD in the L1-L4 lumbar spine (1.088 ± 0.14 vs. 1.109 ± 0.14; *p* < 0.001) when compared with pre-treatment levels [53]. In another study conducted by Ha J. et al. on a group of patients with POI, a significant increase in lumbar spine BMD was observed at one and two years following initiation of HRT compared with a non-HRT control group (*p* = 0.033 and *p* = 0.047, respectively) [54].

When comparing therapy with combined oral contraceptive pills (COCP), it has been suggested that natural 17-β-estradiol (E2) provides superior benefit to synthetic estrogens [41]. In a comparison of a monophasic COCP (Ethinylestradiol 30 µg/Levonogestrel 150 µg daily) with traditional HRT (estradiol 2 mg with addition of levonorgestrel 75 µg for 12 days each month) a significant difference was observed between groups with improvement in lumbar spine BMD at 12 and 24 months observed in the HRT group [55].

Higher estrogen doses (2 mg oral or 100–150 µg transdermal estradiol) have been shown to be significantly more effective at increasing BMD when compared with lower doses or conventional COCPs [41].

Women using combined oral contraceptives containing 30 mg ethinylestradiol continuously for 2 years were found to have a mean increase in BMD at the lumbar spine of 2.5 ± 6.5%, compared with an increase of 1.8 ± 9.9% in a group of women taking high-dose HRT (2 mg estradiol). Furthermore, women treated with low-dose HRT (1 mg estradiol) were found to have a loss in BMD of 1.3 ± 11.5%, while a 3.3 ± 5.4% loss was observed in the untreated control group. These results indicate that treatment with a COCP is effective in increasing BMD as measured at the lumbar spine and total femur and unequivocally more effective than a low-dose estrogen regimen [54]. In 2021, Upton C. et al. presented the POISE study (Premature Ovarian Insufficiency Study of Effectiveness of Hormonal Therapy). This study was designed to compare hormone therapies for superiority using important clinical outcomes as endpoints and monitoring for patient-reported symptoms. The insights gained from this open and pragmatic, parallel, randomized controlled trial is expected to provide a foundation for new POI treatment guidelines in the future [56].

In a recent systematic review, continuous use of combined oral contraceptives was found to have an overall positive impact on BMD. Nevertheless, women in whom loss of bone mass had already started to progress were unable to recover the lost mass despite adequate therapy at common dosages. Additionally, interruption of HRT for any duration longer than one year was linked to significant bone loss [57].

For women with risk factors for venous thromboembolism or stroke, transdermal preparations may be preferred over oral estrogens, as transdermal patches have a lower risk of hypercoagulability by virtue of avoiding the hepatic first-pass effect. Beyond hormone replacement therapy, lifestyle changes should be undertaken in order to minimize associated risk factors and improve BMD. Weight-bearing exercises, smoking cessation, maintenance of normal body weight, and limiting alcohol intake should be recommended to all patients at risk of osteoporosis. Calcium and vitamin D supplementation are equally important. Women who are at a high risk for osteoporosis and who receive adequate calcium from dietary intake alone (approximately 1200 mg daily) are not recommended additional calcium supplementation. Women with inadequate dietary intake, on the other hand, should take supplementary elemental calcium (generally between 500 mg/day and 1000 mg/day), in divided doses with meals, such that their total calcium intake (diet and supplements) is approximately 1200 mg/day [52].

## 8. Prescribing Testosterone

The efficacy of adding testosterone to estrogen replacement therapy in preventing bone loss and improving muscle mass in POI remains unclear. However, testosterone therapy may be indicated for the treatment of hypoactive sexual desire disorder, which may persist despite adequate estrogen therapy [52,58]. Transdermal or non-oral formulations are the preferred route of testosterone administration, as oral testosterone administration has shown to have unpredictable absorption and increased adverse lipid effects [59]. Various formulations, such as transdermal testosterone patches releasing 150 µg/day to 300 µg/day, have been trialed in POI patients.

The 2019 Global Consensus Position Statement on the Use of Testosterone Therapy for Women recommends that while non-oral testosterone therapy be used for women, regular monitoring of serum testosterone levels be performed and should not exceed the physiologic premenopausal range. Unfortunately, due to the pervasive absence of appropriate dosage formulations, testosterone supplementation for women is often not available in many jurisdictions. Should testosterone therapy be initiated, patients should be advised that a noticeable improvement in sexual desire may take several weeks to manifest. If no improvement is noted within 6 months of starting therapy, discontinuation is advised as there is no additional benefit in prolonged treatment [40].

## 9. Non-Hormonal Treatment of POI-Related Osteoporosis

Great progress has been achieved in the development of novel, non-hormonal agents to osteoporosis treatment. It is especially relevant, as we can observe a growing number of patients with contraindications for hormonal therapy, including breast cancer survivors, patients with other, estrogen-based, cancers, and those with venous thromboembolic disease or established cardiovascular disease. Due to the challenge that osteoporosis treatment in these groups can be, new therapeutic options have become available. Bisphosphonates, intranasal calcitonin, parathyroid hormones, raloxifene, risedronate, and strontium ranelate have been proven to decrease the risk of fracture [60].

Bisphosphonates alter the cycle of bone formation and breakdown in the body and are used in osteoporosis prevention and treatment. They have been shown to improve bone mineral density [61]. The mechanism of their action is based on inhibition of bone resorption. Bisphosphonates attach to hydroxyapatite binding sites on the bone, particularly in areas with active resorption, and impairs the osteoclast’s ability to continue bone resorption. Studies have shown that they are capable of reducing bone turnover by up to 90%, which persists throughout the duration of treatment and may reduce the risk of hip fractures up to 30–50% [62]. British Menopause Society guidelines [63] for assessment and management of women with premature ovarian insufficiency recommended that bisphosphonates should not be first-line treatment for the management of osteoporosis in women with POI, which is in line with ACOG recommendations [64]. ACOG underlines that, contrary to the treatment of postmenopausal osteoporosis, which focuses on bisphosphonates as first-line therapy, in women with primary ovarian insufficiency, the most appropriate management of low bone mass is HRT [64]. Bisphosphonates should be considered for the group of POI women with HRT contraindication, for example breast cancer survivors. Hormonal replacement therapy in women with a history of breast cancer is a controversial issue. Therefore, prevention and treatment of osteoporosis in this specific group can be challenging. Being aware of the influence of cancer therapies on bone health and the fact that use of chemotherapy and tamoxifen for young women with breast cancer results in premature menopause in a significant number of patients, it is crucial to ensure bone therapy. The available data suggest an increase of 5% in breast-cancer-related events when hormone replacement therapy is given to women with breast cancer, which creates a limitation to its application in breast cancer survivors [65]. Alternative therapeutic options, including bisphosphonates, have been evaluated to prevent or reverse the bone loss associated with cancer treatments. Majithia et al. [66] investigated a group of postmenopausal women with breast cancer and osteopenia/osteoporosis and concluded that zoledronic acid appears to prevent further bone loss in this population. Additionally, in another study, zoledronic acid has been shown to improve disease-free survival time in breast cancer patients taking anastrozole or tamoxifen and it is claimed to have supplementary adjuvant effects on cancer therapeutics [67]. the main concerns regarding bisphosphonate therapy in POI patients are the safety of this class of drugs and non-clarified influence on fetal development. Resumption of ovarian activity and spontaneous pregnancies are occurring in patients with POI [68]. Moreover, oocyte donation and in vitro fertilization are an established option for reproductive treatment in this group. Being aware that women with POI are able to conceive, long skeletal retention of bisphosphonates can bear the consequences during pregnancy and lactation. As bisphosphonates are incorporated into bone, the terminal half-life is up to 10 years. Stathopoulos et al. [69] reported cases of shortened gestational age, low neonatal birth weight, and transient hypocalcaemia of newborns whose mothers had been exposed to bisphosphonates, as well as few cases of spontaneous abortions and congenital anomalies. Bisphosphonates should be assessed in view of their potential effects on both mother and fetus, and their administration should always be deliberated with the guidance of an osteoporosis specialist [41].

Raloxifene is a selective estrogen-receptor modulator with estrogen-agonist effects on bone and lipid metabolism. It is used for both treatment and prevention of osteoporosis in postmenopausal women. Raloxifene decreases the risk of spine fractures, but not hip fractures, in postmenopausal women with osteoporosis. Raloxifene’s bone effects appear to be mediated through osteoblasts. Raloxifene does not affect the breasts or uterus, and so does not appear to increase the risk of endometrial or ovarian cancer, yet it is crucial to underline the elevated risk of venous thrombosis, including superficial phlebitis, deep vein thrombosis, and pulmonary embolism during raloxifene treatment [70]. Moreover, raloxifene therapy has been found to have a protective effect on the breast against the development of cancer. Raloxifene treatment of postmenopausal women with osteoporosis has shown a decrease in the risk of invasive breast cancer by 76% during 3 years of treatment [71]. So far, the safety of raloxifene in breast cancer patients is unknown, but there seems to be a wide field for research and potential use of raloxifene therapy in breast cancer treatment induce POI.

Denosumab is a human monoclonal antibody with potent antiresorptive activity in bones. It has been shown to increase bone mineral density and reduce fracture risk and markers of bone turnover. It is a receptor activator of nuclear factor kappa-B ligand (RANKL) inhibitor, which prevents activity of RANK expressed on osteoclasts and its precursor cells. Through this mechanism, denosumab reduces the formation, activity, and survival of osteoclasts and inhibits osteoclastic bone resorption. The main concern with denosumab is BMD loss and increased risk of multiple vertebral fractures after discontinuation of treatment. That necessitates follow-on therapy to prevent the previously mentioned negative effects [72]. Denosumab has been shown to reduce vertebral fractures in oncological patients and prevent cancer-therapy-related skeletal issues in breast and prostate cancer [73]. Denosumab is eliminated from the body within several months of administration and so may be safer than bisphosphonates when antiresorptive drugs for osteoporosis are required in POI patients. Studies suggest that denosumab administration during pregnancy or within 5 months prior to conception can result in fetal distress; therefore, 5 month withdrawal is recommended before conceiving [74]. Similarly to bisphosphonates, before starting denosumab therapy, advice from an osteoporosis specialist should be taken [41].

Calcitonin is a naturally occurring anti-resorptive polypeptide hormone secreted by the parafollicular C cells of the thyroid gland that lowers serum calcium levels by decreasing bone resorption and renal tubular calcium reabsorption. Intranasal administration of salmon calcitonin has been used in the management of metabolic bone disorders, including osteoporosis. It has been shown to reduce spinal fracture risk and improve vertebral BMD in postmenopausal women with osteoporosis [75]. Calcitonin is especially effective in individuals with high-turnover osteoporosis, where it provides a net gain of bone mineral in the axial skeleton and a slowing of bone loss in the appendicular bones [76]. Many studies have provided both safety and efficacy data for calcitonin, with local irritating effects of the upper respiratory tract being the most common side effect.

Teriparatide, a parathyroid hormone, acts in a different way than the previously mentioned antiresorptive agents. It is an anabolic drug that can directly stimulate osteoblastic formation of new bone by increasing the number of bone-forming cells, promoting osteoblast growth and decreasing osteoblast cell death or apoptosis [77]. Research has shown that treatment of postmenopausal osteoporosis with parathyroid hormone decreased the risk of vertebral and nonvertebral fractures up to 65% and increased vertebral, femoral and total-body bone mineral density [78]. A potential safety issue with teriparatide is increased risk of osteosarcoma, which was reported in an animal model study [79].

Strontium ranelate is a therapeutic agent with promising effects in osteoporosis treatment, as it is capable of both promoting bone formation and, to a lesser extent, inhibiting bone resorption. It has been shown to decrease the risk of vertebral and nonvertebral fractures and improve bone quality and BMD [80]. Importantly, TROPOS trials indicated that the rate of venous thromboembolism was not increased in the group of women receiving strontium ranelate when compared with placebo [81].

Unfortunately, we are still lacking studies on the clinical utility of calcitonin, teriparatide, and strontium ranelate in POI women [41]. Research on their effects on bone health in this group can create new alternative options for osteoporosis treatment in women with contraindications to hormonal therapy.

## 10. Conclusions

Premature ovarian insufficiency (POI) is a clinically significant condition that affects numerous women every year. It carries a notable risk of decreased bone mineral density (BMD), leading to conditions such as osteopenia, osteoporosis, and low-energy fractures. To address the needs of patients effectively, treatment should be carefully tailored, taking into account individual patient symptoms, preferences, and reproductive goals such as desire for future pregnancy or need for contraception. Properly selected treatment can mitigate the risk of severe complications in many women, underscoring the importance of appropriate therapeutic decisions.
